# In the September 2017 issue

**DOI:** 10.1590/1980-57642016dn11-030001

**Published:** 2017

**Authors:** Ricardo Nitrini

**Affiliations:** Editor-in-Chief

In the September 2017 issue we are publishing one short review, nine original papers, one short communication, one history note, one case report and selected abstracts from the World Congress on Brain, Behavior and Emotions held in Porto Alegre, Brazil, in June 2017.

**Nitrini** reported his study on soccer (football association) and chronic traumatic encephalopathy at the above-mentioned congress. A recommendation to protect soccer players was included.

**Gesualdo et al.** evaluated the cognition of patients with chronic kidney disease on hemodialysis. Most of the patients had low scores on a comprehensive cognitive scale, which were correlated with older age, lower education and longer time on hemodialysis. 

**Morello et al.** carried out a systematic review on non-pharmacological interventions to improve language and communication in Alzheimer's disease (AD). Two intervention techniques emerged as potentially effective.

**Delfino et al.** carried out transcultural adaptation of the Small Communication Strategies Scale developed in Canada for use in Brazil. This instrument evaluates the strategies used by caregivers when communicating with dementia patients and also advises on the best ways to communicate. 

**Guedes et al.** performed a neuropathological study of two cases of frontotemporal dementia, one of which involved amyotrophic lateral sclerosis. Both brains showed TDP-43 deposits, but with distinct topographic distribution.

**Emsaki et al.** evaluated the memory of a small group of patients with amnestic mild cognitive impairment before and after the use of the Memory Specificity Training (MEST). When compared to a control group, the intervention group showed improvement in working and prospective memory.

**Pedraza et al.** performed an epidemiologic study to estimate the prevalence of mild cognitive impairment (MCI) and dementia in adults older than 50 years in Bogotá, Colombia. The prevalence rates for both conditions were higher than those reported in previous studies.

**Cerveira et al.** carried out a systematic review to compare pharmacological and non-pharmacological treatments in elderly patients with delirium. Non-pharmacological interventions were less effective in controlling symptoms of delirium than pharmacological treatments.

**Moraes et al.** performed a systematic review on the acquisition of motor skills (motor learning) in Autism Spectrum Disorder. They concluded that motor learning occurs in this condition although further studies with better design are needed.

**Moura et al.** applied an adapted version of the Mini-Mental State Examination – the Mini-Mental State Examination for Children (MMC) - to screen for cognitive impairments in children with hemiplegic cerebral palsy (CP). They concluded that the MMC is useful as a multiprofessional screening instrument for cognitive impairment in children with hemiplegic CP.

**Contador et al.** investigated the influence of educational level on the progression of cognitive decline in patients with dementia. High-educated patients had more severe decline after a follow-up of about three years.

**Oliveira et al.** reviewed, in a history note, the largely unknown legacy of Francisco de Castro, a Brazilian physician, focusing on his views about the theory of localized cortical functions evident in Europe in the second half of the nineteenth century.

**Iannone et al.** reported an overall improvement of a 78-year-old woman with mild neurocognitive disorder, chronic pain and depression-related symptoms after two 15-day courses of transcranial Direct Current Stimulation (tDCS).

**Baptista et al.** presented a case report of young-onset Alzheimer's disease with preserved awareness of disease, depression and risk of suicide, pointing to the importance of identifying and treating patients with this condition.

**Abstracts from the World Congress on Brain, Behavior and Emotions.** In the 2017 edition of this congress, over 1,000 abstracts were submitted. Of these, 39 abstracts selected by the scientific committee are published here.

**Ricardo Nitrini** Editor-in-Chief

